# Early Transcriptional Response of Soybean Contrasting Accessions to Root Dehydration

**DOI:** 10.1371/journal.pone.0083466

**Published:** 2013-12-12

**Authors:** José Ribamar Costa Ferreira Neto, Valesca Pandolfi, Francismar Corrêa Marcelino Guimaraes, Ana Maria Benko-Iseppon, Cynara Romero, Roberta Lane de Oliveira Silva, Fabiana Aparecida Rodrigues, Ricardo Vilela Abdelnoor, Alexandre Lima Nepomuceno, Ederson Akio Kido

**Affiliations:** 1 Laboratory of Molecular Genetics, Genetics Department, Federal University of Pernambuco, Recife, Pernambuco, Brazil; 2 Laboratory of Genetics and Vegetal Biotechnology, Genetics Department, Federal University of Pernambuco, Recife, Pernambuco, Brazil; 3 Brazilian Enterprise for Agricultural Research – Embrapa Soybean, Londrina, Brazil; 4 LABEX Plant Biotechnology, Agricultural Research Service/United States Department of Agriculture Plant Gene Expression Center, Albany, California, United States of America; Niels Bohr Institute, Denmark

## Abstract

Drought is a significant constraint to yield increase in soybean. The early perception of water deprivation is critical for recruitment of genes that promote plant tolerance. DeepSuperSAGE libraries, including one control and a bulk of six stress times imposed (from 25 to 150 min of root dehydration) for drought-tolerant and sensitive soybean accessions, allowed to identify new molecular targets for drought tolerance. The survey uncovered 120,770 unique transcripts expressed by the contrasting accessions. Of these, 57,610 aligned with known cDNA sequences, allowing the annotation of 32,373 unitags. A total of 1,127 unitags were up-regulated only in the tolerant accession, whereas 1,557 were up-regulated in both as compared to their controls. An expression profile concerning the most representative Gene Ontology (GO) categories for the tolerant accession revealed the expression “protein binding” as the most represented for “Molecular Function”, whereas CDPK and CBL were the most up-regulated protein families in this category. Furthermore, particular genes expressed different isoforms according to the accession, showing the potential to operate in the distinction of physiological behaviors. Besides, heat maps comprising GO categories related to abiotic stress response and the unitags regulation observed in the expression contrasts covering tolerant and sensitive accessions, revealed the unitags potential for plant breeding. Candidate genes related to “hormone response” (LOX, ERF1b, XET), “water response” (PUB, BMY), “salt stress response” (WRKY, MYB) and “oxidative stress response” (PER) figured among the most promising molecular targets. Additionally, nine transcripts (HMGR, XET, WRKY20, RAP2-4, EREBP, NAC3, PER, GPX5 and BMY) validated by RT-qPCR (four different time points) confirmed their differential expression and pointed that already after 25 minutes a transcriptional reorganization started in response to the new condition, with important differences between both accessions.

## Introduction

Soybean [*Glycine max* (L.) Merr.] is recognized as a relevant global crop with an annual contribution to the world economy around US$ 48,6 billion dollars [1], and increasing importance due to its multiple uses in food, feed and industrial applications, such as oil and biodiesel production. In Brazil, soybean represents the main agribusiness product; the country is the largest producer in the world [2]. Despite this status and the fact that soybean is one of the most studied legumes, the soy complex agribusiness has suffered significant losses due to abiotic stresses, with emphasis on drought [3]. In USA, there are reports of around 40% losses caused by water deficit [4], whereas in Brazil, in 2004-2005, soybean severely damaged by drought resulted in approximately 25% yield reduction [5], in an area (southern region) responsible for about 40% of this yield. Last year (2012), in a less severe drought, the production reduced in almost 11% in that same region [6].

Unfortunately, this scenario is increasingly uncertain, considering the climate change perspectives [7]. Therefore, breeding programs looking for effective soybean plants adapted to water deficit are crucial. Studies regarding genetics, physiology and molecular biology of tolerance mechanisms sustaining plant growth and yield under water deficit are essentials for the development of new varieties. In general, features associated with tolerance controlled by many genes make conventional plant breeding more difficult [3,8]. Transcriptome analysis is one of the widest alternatives adopted to identify the repertoire of genes and their biological responses to certain stimuli. Soybean data from various transcriptome projects resulted in a set of 35,986 unigenes [9] stored in GenBank at NCBI (National Center for Biotechnology Information) until May, 2013. Similarly, The Gene Index Project (The Computational Biology Laboratory, Harvard University) includes a total of 137,174 unigenes, consisting of 73,178 TC (Tentative Consensus) sequences, 63,866 singletons and 130 singletons mature transcripts (ET) [10]. Additionally, two microarray slide sets are available; each one consisting of 18,432 single-spotted PCR products derived from the low redundancy cDNA sets [11]. A mixed Soybean GeneChip (http://www.affymetrix.com) is commercially available with ~37,500 *G. max* transcripts, 15,800 *Phytophtora* root and stem root transcripts, and over 7,500 soybean cyst nematode transcripts [3]. Another commercially available microarray platform is the 66 K Affymetrix Soybean Array GeneChip. Despite having high performance, affordable price and still be widely used, microarray technology has serious limitations. Some of them including the cross-hybridization of probes with different potential targets, semi-quantitative results, uncertainty in analysis and interpretation of data, as well as the inability to analyze and discover new genes (only restricted to those immobilized on chips) [12]. 

A recent survey (May, 2013) at PubMed database (NCBI) showed 81 reports related to “soybean and transcriptome”, most of them using microarray approaches, as in the case of Le et al. [13] that used the 66 K Affymetrix Soybean Array GeneChip for genome-wide expression profiling of leaf tissues (soybean cv. Williams 82) subjected to drought stress (soil moisture content of 5% and leaf relative water content = 32±2%) from two stages (V6 and R2). Concerning the reports using high-throughput sequencing methods, Libaut et al. [14] studied the transcriptome of root hair cells under *Bradyrhizobium japonicum* infection. After that, Libault et al. [15] tried to generate a transcriptome atlas using various soybean tissues; Le et al. [16] focused on the NAC transcription factor family in soybean during development and dehydration stress; Li et al. [17], otherwise, looked for stress associated microRNAs in *G. max* by deep sequencing, while Hao et al. [18] searched for soybean genes associated with nitrogen-use efficiency, and Kido et al. [19] looked for plant antimicrobial peptides in soybean transcriptome after *P. pachirizy* induction. Moreover, Fan et al. [20] analyzed the late expression (48 h after stress) to different conditions including drought (2% PEG 8000), in leaves and roots of seedlings (two-leaf stage) of the soybean inbred line HJ-1. Using RNA-Seq method, specifically, Severin et al. [21] searched for a high-resolution gene expression in a collection of fourteen different tissues; Hunt et al. [22] tried to characterize the transcriptional profiles of a wild-type and glabrous soybean lines while Reid et al. [23] looked for transcript abundance changes that occur during AON (autoregulation of nodulation), and Peiffer et al. [24] attempted to identify candidate genes underlying an iron efficiency quantitative trait locus. 

Thus, it is clear that there is still a gap in regard to reliable information on transcriptomics to recognize the initial response to water deficit response in soybean. Also, no previous transcriptome approaches evaluated contrasting (tolerant/sensitive) soybean accessions. Thus, the aim of this study was to fill this gap using DeepSuperSAGE (26 bp tags), a highly sensitive transcriptome method, comparing contrasting accessions under root dehydration stress (25-150 min), aiming to identify tolerance-associated gene candidates, especially regarding the early response not evaluated up to date. 

## Results and Discussion

### Qualitative and Quantitative Analysis of the DeepSuperSAGE Libraries

The DeepSuperSAGE libraries based on the total number of sequenced tags [2,551,286, of which 1,030,443 for ‘Embrapa 48’ (tolerant accession) and 1,520,843 for ‘BR 16’ (sensitive accession)] allowed a comprehensive evaluation of the soybean transcriptome under root dehydration stress. Thus, after singlets exclusion from the total number of tags, 120,770 unitags (unique tags) followed for further analysis. Comparing the contrasts between two libraries, the unitag number ranged from 73,807 to 89,205 (Table 1). It should be highlighted that the estimated number of protein-coding loci for soybean is 66,153 [25]. Thus, the high number of unitags (120,770 for the four libraries) could be justified by the presence of sister unitags (those with a single base difference in a given position and not grouped in a consensus unitag), possibly constituting potential SNPs, alternative transcripts or (less probably) artifacts. 

**Table 1:** Number of differentially expressed soybean unitags (UR: u-regulated; DR: down-regulated; n.s.: non-significant at *p* < 0.05) based on SuperSAGE libraries contrasts.

	**ET1-6 *vs* ET0**		**BT1-6 *vs* BT0**		**ET1-6 *vs* BT1-6**		**ET0 *vs* BT0**	
	**Tags**	%	**Tags**	%	**Tags**	%	**Tags**	%
UR	13,532	18.1	10,751	12.0	12,347	16.7	6,468	7.9
DR	7,423	9.9	5,587	6.3	7,634	10.3	3,135	3.8
n.s.	53,878	72.0	72,867	81.7	53,826	73.0	73,067	88.3
Unitags	74,833	100.0	89,205	100.0	73,807	100.0	82,67	100.0

*ET0 (tolerant accession ‘Embrapa 48’; unstressed control); BT0 (sensitive accession ‘BR 16’; unstressed control); ET1-6 (‘Embrapa 48’ after root dehydration stress); BT1-6 (‘BR 16’ after root dehydration stress).

The number of differentially expressed up- (UR) and down-regulated (DR) unitags and those not differentially expressed (n.s.), at the level of *p* < 0.05 (see Material and Methods), for some contrasting libraries can be seen in Table 1. The n.s. unitags accounted for more than 70% of the total, regardless of the considered contrast (Table 1), and probably regard housekeeping genes or genes associated to other physiological processes. Otherwise, the number of UR unitags was higher than the DR in all contrasts (Table 1), also when comparing both accessions under stress (ET1-6 *vs* BT1-6) and even both negative controls (ET0 *vs* BT0; Table 1). 

### Primary Annotation of DeepSuperSAGE Unitags

After annotation (BLASTn) of the 120,770 unitags against different EST databases, 57,610 (47.7%) of them presented ESTs matches tolerating a single mismatch (TSM) maximum in the alignments (Table 2). From those TSM alignments, 32,373 unitags (56.2%) could be annotated based on previous characterized ESTs (Table 2), disregarding the “unknown” hits (ESTs, cDNAs or mRNAs) or just clones or chromosomes annotations with no given function. Concerning the annotated unitags, 14,903 (46.0%) of them showed 100.0% identity (26 bp of the unitag) in perfect BLASTn alignments with ESTs (Table 2), which 14,545 of them with *G. max* ESTs (data not shown). Such ESTs, when related to differentially expressed unitags, are potentially useful for primer and probe design, aiming RT-qPCR validation and, at the same time, avoiding following sequencing for unitag identification. Alternatively, from those unitags with appropriate ESTs (57,610), it was possible to characterize 35,985 unitags by GO (Gene Ontology), i.e., more expressive than those 32,373 unitags associated with ESTs with appropriate annotations (Table 2). Thus, for those unitags aligned to ESTs without a decent gene/function description, the GO characterization was a valuable reference and information source.

**Table 2:** Summary of primary annotation of the unitags.

**Features**	**Alignment unitag-EST**		**Total**	**%**
	**Single mismatch**	**Perfect**		
Unitags	-	-	120,770	100.0
Unitags with no hit	-	-	63,160	52.3
Unitags with hits	26,911	30,699	57,610	47.7
With descriptions	17,470	14,903	32,373	56.2^*^
Without description	9,441	15,796	25,237	43.8^*^
With GO terms	18,619	17,366	35,985	62.5^*^

^*^ In relation to 57,610 (unitags with hits).

Regarding perfectly aligned (100% identity) unitags with ESTs (Table 2), 15,796 remained non-annotated. Those unitags and appropriate ESTs can be a valuable source of candidates for further evaluations and inferences on their function, especially concerning those differentially expressed and responsive against root dehydration stress. On the other hand, besides the 14,903 unitags presented ESTs descriptions, others 17,366 unitags showed EST-GO terms (Table 2). The best characterized set of unitags (i.e., adequate annotation and carriage GO terms) comprised 24,924. Another appealing group (9,441 unitags with a single mismatch; Table 2) showed ESTs with no informative descriptions, requiring further characterization. A third useful group comprised 63,160 unitags with “no hit” after BLASTn (Table 2). These numbers emphasize the importance of the DeepSuperSage open architecture technology, allowing access to new gene-candidates.

### Anchoring of Unitags in Soybean Genome

**Figure 1:**
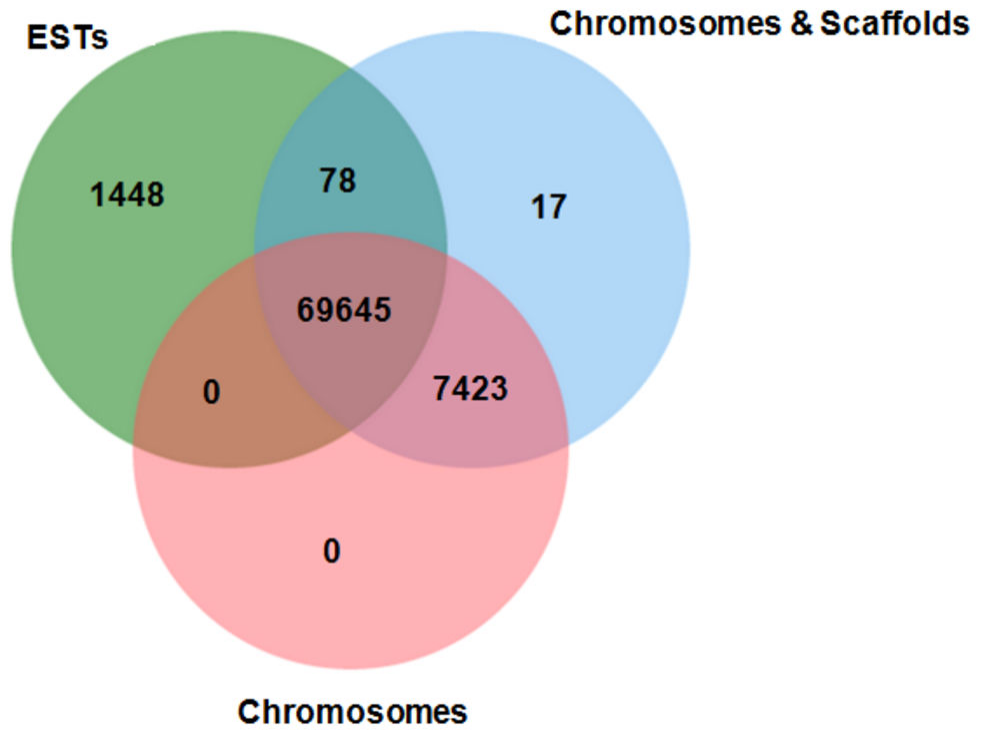
Venn diagram for sets of soybean unitags aligned* with soybean ESTs and genomic sequences**. * Via BLASTn (tolerating maximum of one mismatch). ** Soybean genome browser (Phytozome database: http://www.phytozome.net/).

The unitags aligned via BLASTn against soybean transcripts and genome, both from the Phytozome database (http://www.phytozome.net/), allowed the identification of potential non-annotated genes. The BLASTn analysis involved in TSM alignments of unitags - ESTs comprised 71,171 unitags and 44,204 ESTs, which was restricted to 27,190 unique ESTs, when only the best hits were considered. On the other hand, the BLASTn analysis against the soybean genome included TSM alignments, ending up with 77,163 anchored unitags in 20 chromosomes and some scaffolds (data not shown). In an effort to determine which unitags were present in each group (ESTs, chromosomes or scaffolds), a Venn diagram (Figure 1) showed that, from the 71,171 aligned unitags with the soybean ESTs, 78 were also anchored in scaffolds, while 69,645 were anchored in chromosomes, as well. This result is consistent with what it was expected since the DeepSuperSAGE tags are generated mainly from the 3’UTRs presented in both genomic and transcripts sequences. Moreover, 1,448 unitags (Figure 1) aligned only with ESTs. When analyzing in which transcript region these alignments took place, almost all of them (1,290) showed match (TSM) with coding regions (CDS; data not show). This was not a predicted outcome. Once the *Nla*III is a frequent cutting enzyme, it was expected the digestion in 3'UTR of each expressed transcript, as mentioned before. A possible explanation for these results could be a partial digestion of cDNAs by the *Nla*III enzyme. In an attempt to minimize it, the cDNAs underwent a process of double digestion by the enzyme. Nevertheless, a lack of the *Nla*III enzyme restriction site in the 3’UTR or an unforeseen gene transcript sampling or even an alternative, non-described, transcript for an already predicted gene, could all explain these generated unitags. 

 Besides, 7,440 unitags exclusively anchored in genomic regions, without any ESTs alignments, with almost all of them (7,423 unitags) anchoring in the predicted chromosomes (only 17 anchored in scaffolds; Figure 1). From this total of chromosomes anchored unitags, 1,865 were differentially expressed in the stressed *vs* control contrast involving tolerant or sensitive accession (data not shown). To almost all of them (1,667) it was observed the perfect match of the unitags (26 bp) with the genomic sequences (data not shown). These results can indicate the presence of genes in those regions or of new transcripts that were sampled, or even of alternative, non-described, already predicted genes, but all these possibilities would include transcripts that have significant responses to the applied stress. 

**Figure 2:**
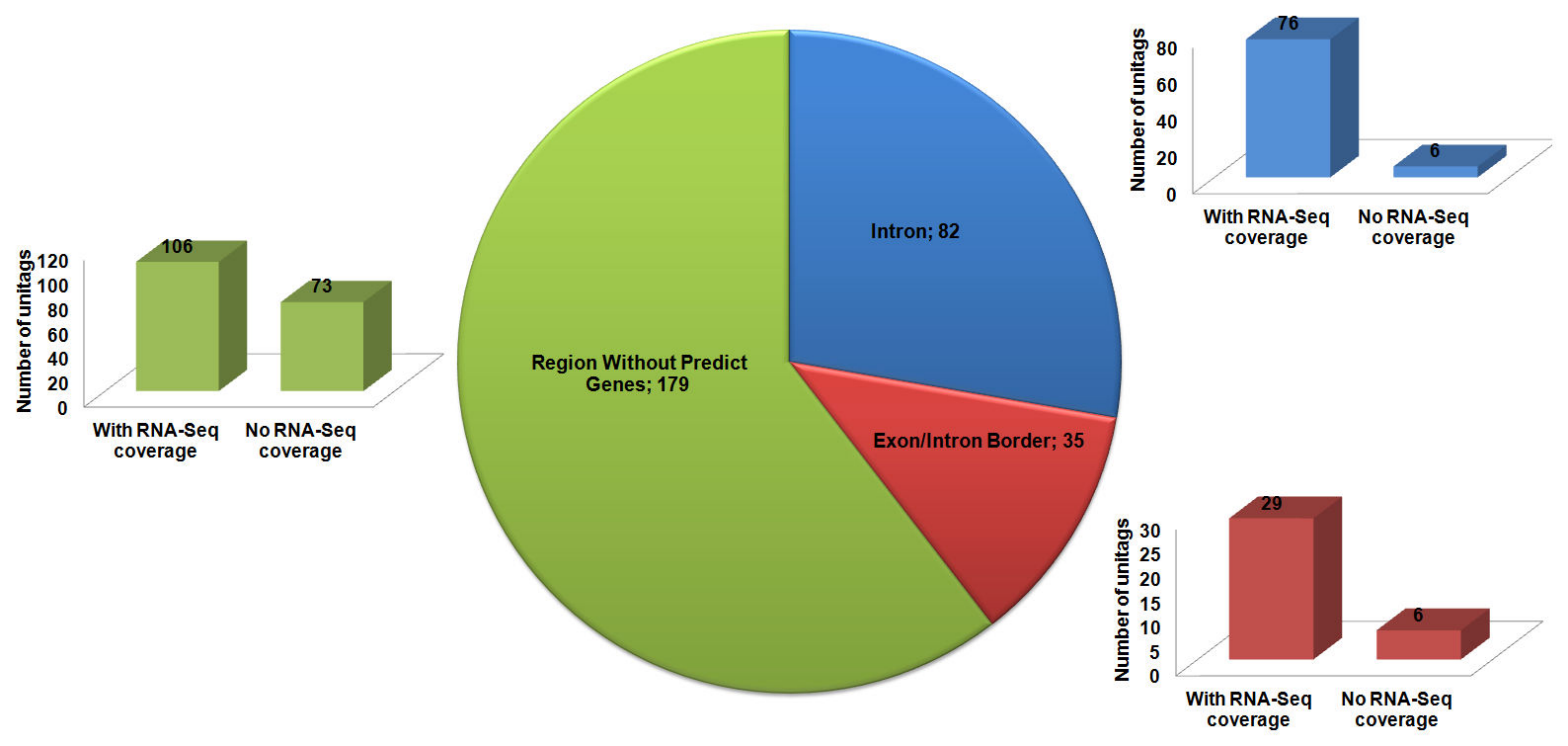
Number of unitags anchored in different soybean genomic regions*, with or without the coverage of RNA-Seq data*. * According to the soybean genome browser (Phytozome database: http://www.phytozome.net/).

**Figure 3:**
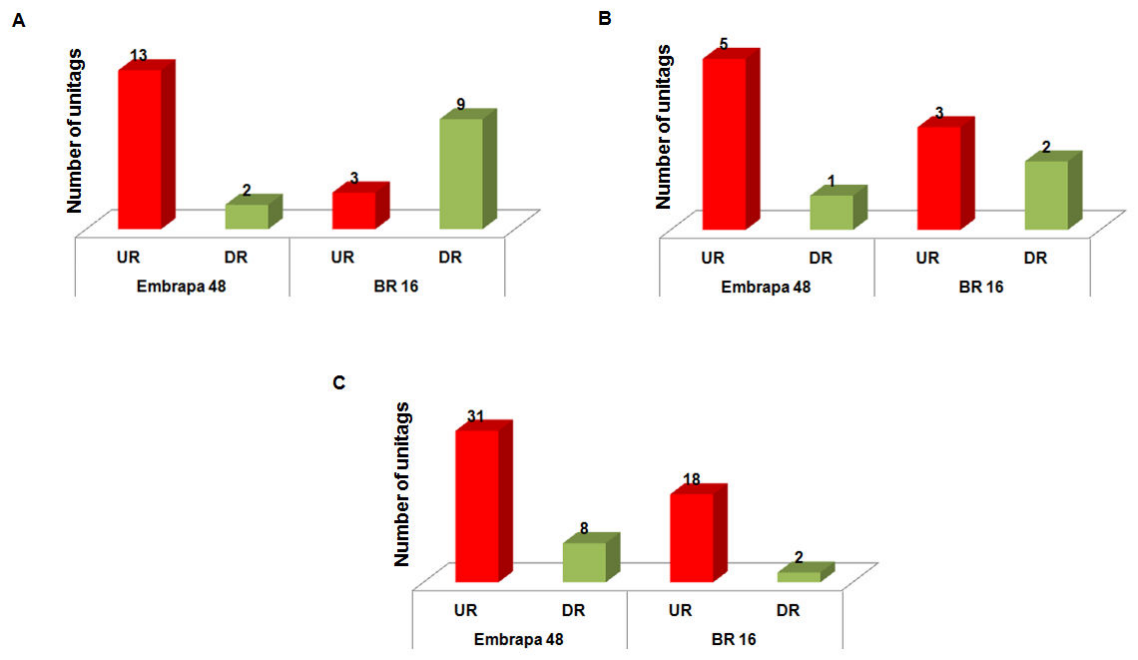
Number of unitags differentially expressed for each accession, and mapped in genomic regions* with predicted coverage by RNA-Seq*. (A) unitags mapped in introns; (B) unitags mapped in exon/intron borders; (C) unitags mapped in genomic regions without any predicted gene. * According to the soybean genome browser (Phytozome database: http://www.phytozome.net/). UR: up-regulated; DR: down-regulated.

 A more detailed analysis comprising 296 unitags anchored to chromosome 1, using the tool genome browser at the Phytozome site, showed 82 unitags anchored at introns and another 35 at the exon/intron borders (Figure 2). To the majority of these anchored sites, gene expression was reinforced by available RNA-Seq data, as indicated in the genome browser (Figure 2). Additionally, 179 unitags anchored in regions without predicted genes in their surroundings, notwithstanding 106 of those unitags presented in their respective loci RNA-Seq data covering it (Figure 2). In those sites covered by the RNA-Seq, differentially expressed unitags (*p* < 0.05) were observed after the stress stimulus (Figures 3A, 3B and 3C). Regarding this differential gene expression response, Embrapa 48 showed more induced unitags than its counterpart BR 16 (Figures 3A, 3B and 3C). Thus, the DeepSuperSAGE data, in association with the RNA-Seq data mentioned for those unannotated regions of the soybean genome, suggest that those regions may play important roles in the plant physiology, acting in response to root dehydration and assisting in plant homeostasis maintenance. Meanwhile, more studies are needed to determine the real importance of these sequences in the analyzed stress response. 

### Distribution of the Differentially Expressed DeepSuperSAGE Unitags

For a better understanding of the contrasts between libraries, it is necessary to understand the effects included in each comparison. The ET1-6 *vs* ET0 contrast (approach I) addressed the drought-tolerant response to root dehydration; BT1-6 *vs* BT0 (II), the drought-sensitive response to the stress; ET0 *vs* BT0 (III), the differences between the accessions under normal conditions (controls), and ET1-6 *vs* BT1-6 (IV), the differences when both accessions were under stress.

**Figure 4:**
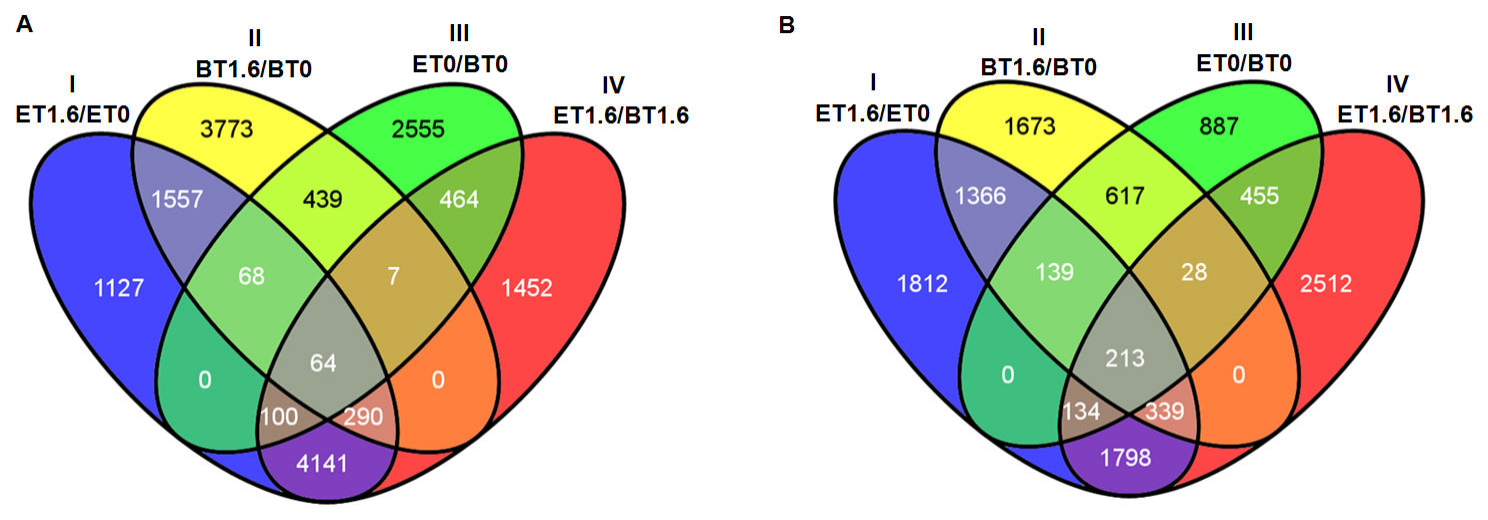
Venn diagram of the UR (A) and DR (B) unitags from soybean DeepSuperSAGE libraries. UR: up-regulated; DR: down-regulated; I-Tolerant accession under stress *versus* respective control (ET1.6, tolerant accession under stress library; ET0, tolerant accession control library); II-Sensitive accession under stress *versus* respective control (BT1.6, sensitive accession under stress library; BT0, sensitive accession control library); III-Tolerant accession control library *versus* sensitive accession control library; IV-Tolerant accession under stress library *versus* sensitive accession under stress library.

Considering the UR unitags, a Venn diagram (Figure 4A) isolated 1,127 unitags only observed in the drought-tolerant accession response to the stress (approach I), in contrast to 3,773 unitags only observed in the sensitive accession response (approach II), and while 1,557 unitags showed induction in both accessions. These exclusive UR unitags from the drought-tolerant accession probably included those transcripts and genes responsible for a better performance of this accession under the stress applied. The annotation of these tolerant-exclusive UR unitags showed 484 with informative descriptions (gene/function) and GO terms associated while 162 presented only descriptions, 209 only GO terms and 272 with no information regarding their role (Table 3). 

**Table 3:** Number of soybean UR unitags presented in different comparisons.

	**Exclusive UR unitags**	**Common UR unitags**
**Descriptions / GO terms**	(ET1.6 *vs* ET0^*^)1	(ET1.6 *vs* ET0^*^)^1^ & (ET1.6 *vs* BT1.6^*^)^2^
With description / with GO	484	1,734
With description / no GO	162	561
No Description / with GO	209	809
No Description / no GO	272	1,037
**Total**	1,127	4,141

UR (up-regulated); *ET0 (tolerant accession ‘Embrapa 48’; unstressed control); BT0 (sensitive accession ‘BR 16’; unstressed control); ET1.6 (‘Embrapa 48’ after root dehydration stress); BT1.6 (‘BR 16’ after root dehydration stress).

Another useful set regarded the 4,141 UR unitags shared by the approaches I and IV (Figure 4A) that highlight the differentially induced expression of the drought-tolerant accession under stress as compared with the appropriate negative control or the sensitive accession also under stress. Considering these unitags, 1,734 presented informative descriptions and GO terms associated, while 561 only presented descriptions, 809 only GO terms and 1,037 lacked any knowledge (Table 3). A third relevant set included 290 unitags over-expressed in I, II and IV (Figure 4A), regarding UR unitags in the respective approaches: tolerant, sensitive, and both accessions under stress response. 

The same evaluation may be carried out in the DR unitags (Figure 4B). In this analysis, 1,812 unitags showed suppression exclusively in the drought-tolerant accession response to root dehydration (approach I) while 1,798 presented in approaches I and IV (Figure 4B). From these 3,610 DR unitags (1,812 + 1,798), 1,691 presented adequate descriptions and GO terms, while 421 presented only descriptions; 766 only GO terms, whilst 732 remained uncharacterized (data not shown). Another group (339 DR unitags, Figure 4B) showed DR unitags in the approaches I (tolerant), II (sensitive), and IV (both accessions under stress). The real meaning of these suppressed sets should be investigated.

The high number of promising candidates based on unitags highlights the potential of the DeepSuperSAGE technology in the disclosure of relevant transcripts responding to the applied stress. The first step to understand the functional background relies on the use of bioinformatic tools and the effective annotation and functional categorization of the differentially expressed unitags.

### Functional Categorization of ESTs Anchoring DeepSuperSAGE Unitags

**Figure 5:**
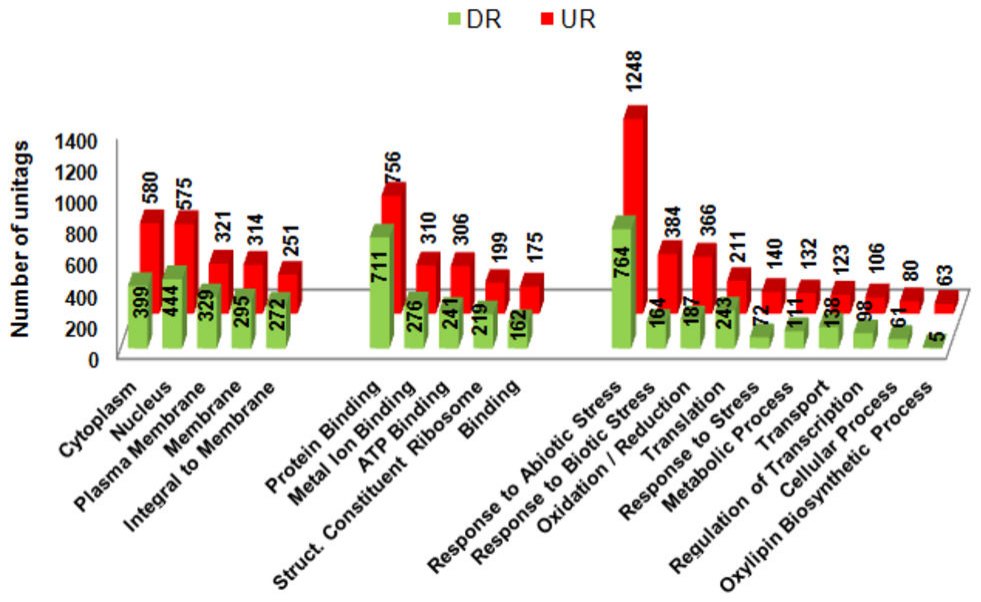
Gene Ontology categorization of the tolerant accession transcripts based on the UR^*^ and DR^*^ soybean DeepSuperSAGE unitags. UR: up-regulated; DR: down-regulated ; *Unitags from tolerant accession under stress versus respective control.

The GO categorization [26] of 42,042 ESTs related to the unitags resulted in 179,670 different terms, including the three main categories: Biological Process (BP; 67,459), Molecular Function (MF; 61,568) and Cellular Component (CC; 50,643). The categorization of the ESTs related to the drought-tolerant accession (Figure 5) considered the GO terms regarding the 8,634 differentially expressed unitags (65.7% of all UR and DR unitags, approaches I, Figure 4A and 4B, respectively). The CC category refers to the place in the cell where the gene products are working [27]. The most represented CC subcategories were: “nucleus” (GO: 0005634; 575 UR and 444 DR tags), “cytoplasm” (GO: 0005737; 580 UR and 399 DR unitags) and “plasma membrane” (GO: 0005886; 321 UR and 329 DR unitags) (Figure 5). The expected prevalence of these cell compartments represent the lodging site of the genetic material responsible by the coordination of their cellular functions and reactions, and also because cell membranes are the first stress receptors, protecting the cell from modifications affecting both stress perception and rigidity of the cell structure [28]. For instance, a change in the fluidity of the plasma membrane might induce a conformational change in a receptor that activates a downstream kinase cascade [29]. Furthermore, cellular membranes threatened by reactive oxygen species (ROS) during cell metabolism, as a result of stress [30-32], produce lipid peroxides that can be used as a stress indicator [28].

Regarding the MF categories, the terms most represented was “protein binding” (GO: 0005515756; 756 UR and 711 DR unitags), “metal ion binding” (GO: 0046872; 310 UR and 276 DR unitags) and “ATP binding” (GO: 0005524; 306 UR and 241 DR unitags) (Figure 5). “Protein binding” represents selective and non-covalent interactions with any protein or protein complex, including binding to calcium-dependent proteins, calmodulin receptors, and transcription factors, among others. Considering those descriptions, the importance of this category can be seen in a brief summary where abiotic stresses (mainly drought and salinity) induce changes in cytosolic Ca^2+^ levels [33]. Ca^2+^-binding proteins [calcium-dependent protein kinase (CDPK), calmodulin (CaM), and calcineurin B-like protein (CBL)] serve as transducers of the Ca^2+^ signal, leading to the activation of the signaling pathways, resulting in plant responses to those stresses [34-36]. These Ca^2+^-binding protein classes were expressed in all the evaluated contrasts (data not shown), whereas CDPKs and CBLs presented a higher number of up-regulated unitags when comparing both stressed accessions in relation to the appropriate negative controls (Table S1). CDPKs have recognized participation in abiotic stress tolerance, especially in the modulation of ABA signaling to reduce ROS [37]. In turn, CBLs showed an association with drought tolerance and osmotic stress in Arabidopsis. Loss-of-function Arabidopsis mutants lacking CBL1, CBL9, or CIPK1 found to be more sensitive to drought and osmotic stress than the wild-type plants [38,39].

 In turn, “metal ion binding” represents proteins that interact selectively in a non-covalent way with any metal ion. In this way, dehydrins, considered effective in the tolerance process to different stresses [40], present metal binding properties to Fe^+3^, Co^+2^, Ni^+2^, Cu^+2^ and Zn^+2^ [41]. Considering the contrasting accessions after stress, a total of 101 unitags associated with this protein family could be identified. Interestingly, the sensitive accession had an increased number of up-regulated unitags (70) than its tolerant counterpart (50) (Table S2). Considering UR unitags in the tolerant accession, 24 of them were n.s. or non-observed in the sensitive accession (Table S2), becoming potential targets for further studies.

“ATP binding” includes proteins that interact selectively and in a non-covalent way with ATP (adenosine 5'-triphosphate), a universally relevant coenzyme and enzyme regulator. Among them, ABC transporters stand out, also for their involvement in the abscisic acid (ABA) transport [42]. This stress-related hormone plays a key role in the tolerance process against abiotic stresses, especially regarding drought and salinity [43]. In the present evaluation, 23 possible ABC transporters found to be differentially expressed in the analyzed accessions, being eight induced only in the tolerant accession (Table S3). 

In general, the analyzed transcripts in both accessions showed similar isoforms regulation based on unitags, but some presented contrasting regulation (e.g. UR in the tolerant and DR/n.s. in the sensitive) or accession-specific unitags (Table S1, S2 and S3), and these may act in their physiological differentiation when the drought stress is applied. Concerning the BP categories (biological processes in which the gene products are involved [27]), the two most depicted subcategories were “response to abiotic stress” (GO: 0009628; 1248 UR; 764 DR unitags; Figure 5) and “response to biotic stress” (GO: 0009607; 387 UR; 164 DR unitags; Figure 5). Further details will be address in the next topic, due to the importance and pertinence of the “response to abiotic stress” to the current evaluated subject (root dehydration). The second well represented subcategory was “response to biotic stress” that highlights the crosstalk mechanism, i.e., the co-activation of genes among both biotic and abiotic stress types. For example, the interaction of transcriptional regulation of environmental challenges, such as heavy metal (CuSO_4_) stress, with incompatible necrotrophic pathogen infection revealed significant overlap between biotic and abiotic stress responses [44]. Also, large-scale microarray transcriptome data strongly supported the existence of such interaction between signaling networks [45]. Moreover, the reactive oxygen species (ROS) generation is as a key process shared between biotic and abiotic stress responses [46,47]. Thus, a growing number of evidences supports the notion that plant signaling pathways consist of complex networks with some crosstalk, thereby allowing plants to regulate both abiotic stress tolerance and disease resistance. 

### Analysis of the GO Subcategory “Response to Abiotic Stress”

The expression patterns by heats maps of differentially expressed unitags related to “abiotic stress response” GO category, considered different contrasts and the modulation expression values (FC) of such unitags. This GO category included “response to hormone stimulus” (GO: 0009725), “response to water” (GO: 0009415), “response to salt stress” (GO: 0009651) and “response to oxidative stress” (GO: 0006979). 

### Response to Hormone Stimulus

Hormones are chemical messengers that trigger different processes in animal development, being also present in the vegetal kingdom controlling various aspects of plant growth and development [48]. Plant hormones [salicylic acid (SA), jasmonic acid (JA), ethylene (ET) and ABA] form a complex system that plays key roles in disease resistance and response to abiotic stresses, including drought [49,50]. 

**Figure 6:**
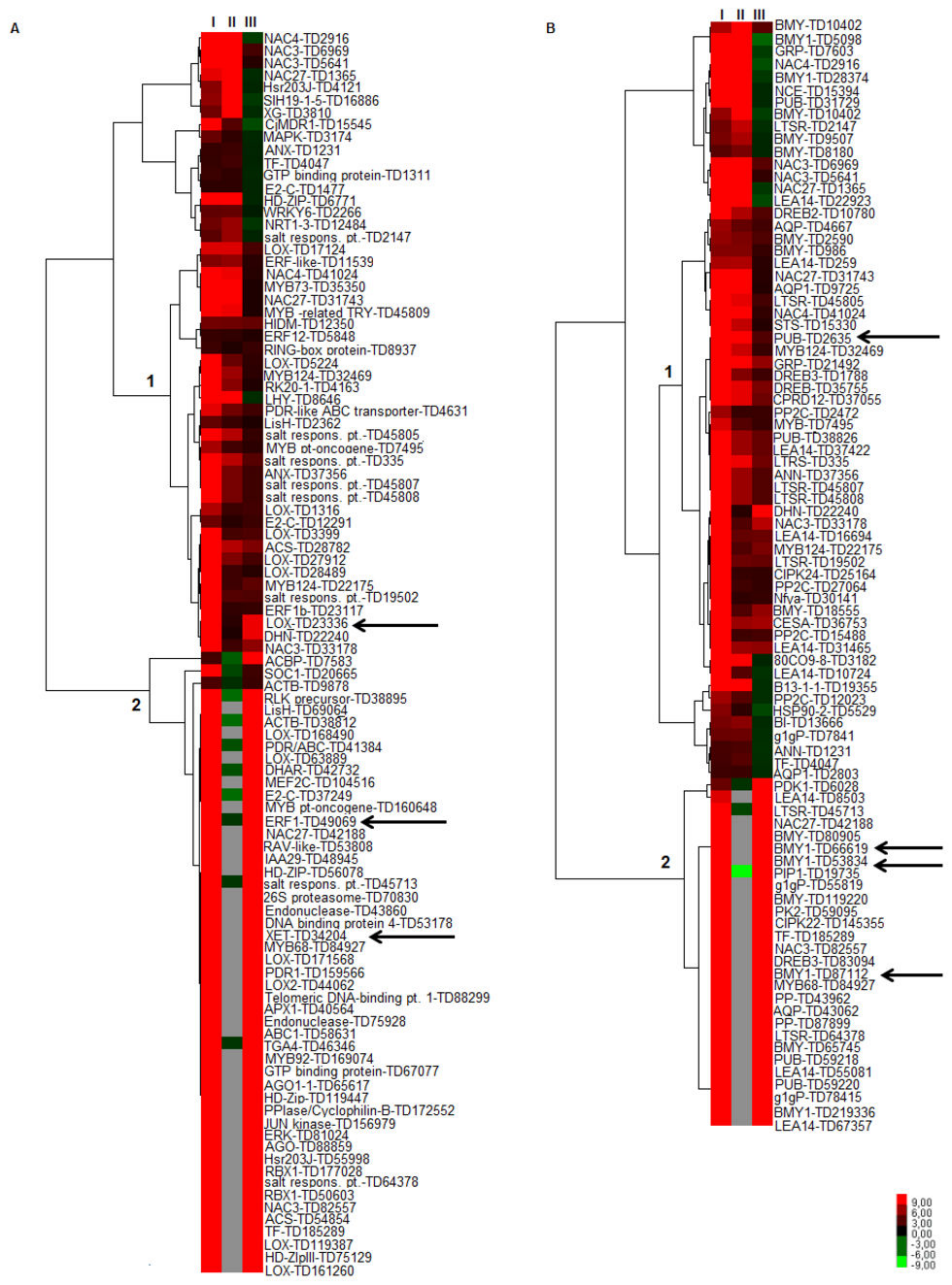
Hierarchical clusterization^1^ regarding GO categories [(A) Response to hormones; (B) Response to water], and several contrasts^2^ ^1^Gray spots: no expressed unitags; Red: up-regulated unitags; green: down-regulated unitags; black: constitutive expression ^2^. Tolerant accession under stress vs. respective control (I); Sensitive accession under stress vs. respective control (II); and Tolerant accession vs. Sensitive accession, both after root dehydration stress (III). Arrows indicate transcripts mentioned in the discussion.

The UR unitags clusterization covering “response to hormone stimulus” and the tolerant response (approach I) compared with the sensitive one (approach II) or even both accessions under stress (approach III) resulted in a heat map (Figure 6A; Table S4) where clusters 1 and 2 (left side of the heat map) might be highlighted. The Cluster 1 (Figure 6A; Table S4) regards unitags presented in both accessions and co-induced mainly in approaches I and II, also some unitags in III. Such a similar expression even in contrasting accessions may represent a key role of such genes in the process of acclimatization to the additional condition imposed. Example of this group was a lipoxygenase, with meaningful FCs [LOX, TD23336; FC_I_ = 36.0, FC_II_ = 1.5, FC_III_ = 8.5] (Figure 6A). LOX is an enzyme implicated with developmental processes and responses to stress and hormones in plants. Bell and Mullet [51] observed water deficit response associated with overexpression of some LOX isoforms in soybean (*G. max*) and pea (*P. sativum*).

 The Cluster 2 (Figure 6A; Table S4) covered UR unitags in the tolerant response (I and III) and DR or absent in the sensitive response (II). Such unitags may be associated with genes whose regulation helps in the distinction of physiological behavior among accessions. Representative of this group was an Ethylene-responsive transcription factor 1b (ERF1b, TD49069; FC_I_ = 9.6, FC_II_ = -1.9 and FC_III_ = 9.6), a gene known for its activation in response to ethylene hormone. The expressions of ethylene-related genes occur through transduction of the ethylene signal from receptors to dedicated transcription factors [52]. ERFs (restricted to plants), an AP2/EREBP-type transcription factors, which serve as trans-acting factors at the last step of transduction [53], presented implications on stress tolerance against abiotic stress. An ERF protein, JERF3, overexpressed in tobacco was responsible for a better adaptation to stresses, such as water deficit, freezing and high salinity [54]. The same authors observed the transcription factor activity on the control of genes involved in the oxidative stress regulation. The overexpression of another ERF gene in rice (TSRF1) also increased the tolerance against drought [55]. In the same way, a putative transcription factor ERF1b (UR in our soybean libraries), usually correlated with basal metabolic processes (development and fruit ripening in plum) [56], showed results indicating a possible involvement in soybean root dehydration response. Also, two AP2/EREBP transcripts, with expressions validated by RT-qPCR (see the specific item), reinforced this association. Another highly modulated unitag was associated to the xyloglucan endotransglycosylase family (XET, TD34204; FC_I_ = 26.4, FC_III_ = 26.4). Enzymes of this family have the potential to enzymatically modify wall components modulating the degree of cross-linking in the cell wall to allow cells to expand during development [57]. The first molecular genetic evidence that connects the cell wall and plant stress tolerance was provided after overexpression of a cell wall peroxidase in tobacco, improving the seed germination of transgenic plants under osmotic stress [58]. Other putative XET transcript isoform unitag (TD31210) analyzed by RT-qPCR, in the present work, validated the contrasted expression showed by the accessions (see the results along this article). 

### Response to Water

 Unitags associated to the GO “response to water” and up-regulated in the approach I, when compared with the *in silico* expression in the approaches II and III, regarding their modulation of expression, presented, as in the previous situation, two clusters (1 and 2, Figure 6B). The modulation of the respective genes highlighted in that clusters may be explained in accordance with the reasoning presented in Figure 6A. 

 The Cluster 1 (Figure 6B; Table S5) showed unitags available in both accessions and up-regulated in most of the three approaches (I, II, and some of the III). This was the case of the putative U-box E3 ubiquitin ligase (PUB, TD2635) that showed one of the highest frequency modulation (FC_I_ = 45.0, FC_II_ = 17.3, FC_III_ = 2.2). Such protein is a part of the ubiquitin-proteasome (Ub-26S) pathway, a cascade mediated by three sequential ubiquitination enzymes that modify the selective ubiquitin ligation. About more than 5% (> 1,300 genes) of the Arabidopsis genome encodes main components that operate in the Ub-26S pathway, where about 1,200 genes encode for E3 ubiquitin ligase components [59]. This abundance illustrates how valuable this protein degradation process is in plants. The large number of E3 ubiquitin ligase genes relative to the Ub pathway-related genes in Arabidopsis and other eukaryotes is indicative of the importance of the E3 ubiquitin ligase step during the selectivity of the ubiquitin-proteasome pathway. Some induced isoforms present intimate relationship with abiotic stress, especially water stress, acting as negative regulators in Arabidopsis, coordinately controlling a drought signaling pathway by ubiquitinating cytosolic RPN12a [60]. 

 In turn, the cluster 2 (Figure 6B; Table S5) includes unitags potentially valuable in the tolerance response. The three most expressed unitags in this group (TD87112, TD53834 and TD66619), annotated as β-amylase (BMY) enzymes, showed FCs (FC_I_ and FC_III_) ranged from 19.2 to 24.0 (Table S5). BMY expression and activity is affected by abiotic stress including osmotic stress and drought. Exposure of barley [61], pearl millet and maize [62] to osmotic stress (300 mM sorbitol for four days) resulted in the increase of vacuolar BMY activity and BMY protein levels. Similarly, when cucumber cotyledons treated with 30 or 50% polyethylene glycol for up to one day, BMY activity increased followed by increases in sucrose and maltose [63]. Yang et al. [64], in turn, observed that both α- and β-amylase activities were enhanced by water stress, with the former enhanced more than the latter, and were significantly correlated with the concentrations of soluble sugars in the stems. It has been suggested that these sugars work in the osmotic adjustment process in plants [65].

### Response to Salinity

**Figure 7:**
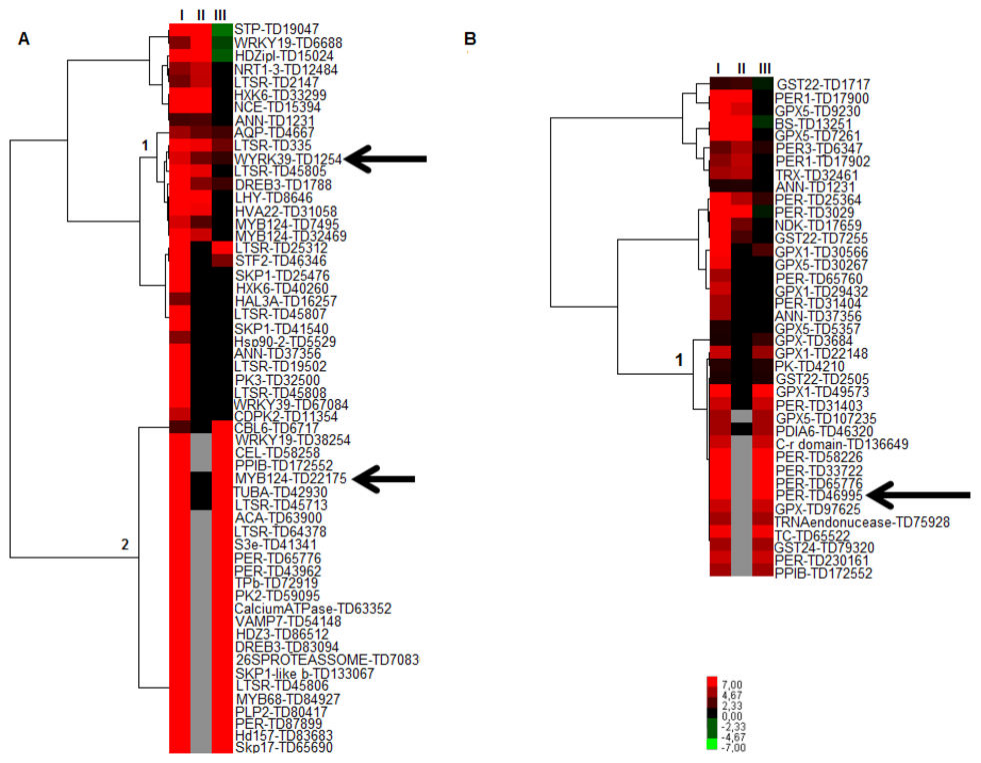
Hierarchical clusterization^1^ regarding GO categories [(A) Response to salinity, (B) Response to oxidative stress], and several contrasts^2^ ^1^Gray spots: no expressed unitags; Red: up-regulated unitags; green: down-regulated unitags; black: constitutive expression ^2^. Tolerant accession under stress vs. respective control (I); Sensitive accession under stress vs. respective control (II); and Tolerant accession vs. Sensitive accession (both under stress; III). Arrows indicate transcripts mentioned in the discussion.

The exposure to drought or salt stress triggers many common reactions in plants. Both stresses lead to cellular dehydration, which causes osmotic stress and water removal from the cytoplasm into the extracellular space, resulting in a reduction of the cytosolic and vacuolar volumes [66]. Early responses to water deficit and salt stresses are largely identical, except for the ionic component. These similarities include metabolic processes, such as photosynthesis [67] and hormonal processes, like rising levels of the plant hormone ABA [68]. Those processes include genes potentially involved in the crosstalk response. The heat map, comprising unitags associated to the GO category “response to salinity” and up-regulated in the tolerant response, when compared with those observed in the approaches II and III, highlighted two distinct clusters (Figure 7A). 

The Cluster 1 (Figure 7A; Table S6) encloses unitags presented in both accessions, but UR in the approaches I, II and III, or even n.s. in the approach III. The up-regulation in approach III regarded unitags with a higher expression in the tolerant after stress, when compared with the sensitive one. This situation regarded TD1254 unitag (FC_I_ = 6.1, FC_II_ = 3.1 and FC_III_ = 1.5), a possible WRKY transcription factor. According to Eulgem et al. [69] members of this family were overexpressed responding to various stress types. Among 72 WRKY genes (Arabidopsis), 49 presented differential expression in response to hormones (salicylic acid treatment) or biotic stress (infection by a bacterial pathogen) [70]. Also, these genes were implicated in responses to wounding (*A. thaliana* [45]), drought and heat (tobacco [71]) and cold (*Solanum dulcamara* [72]). At least 64 soybean SuperSAGE unitags were possible WRKY transcription factors transcripts (data not show). 

The Cluster 2 (Figure 7A; Table S6) contain unitags induced in approaches I and III (absent or n.s. in the approach II). One of the most expressed modulated unitags in this group was TD22175 (FC_I_ = 28.8, FC_III_ = 3.4), a possible MYB transcription factor. Members of the MYB family are abundant in all eukaryotes, being the most frequent transcription factor family (TF) in plants [73]. In the present data, 425 unitags annotated as MYB TFs (data not show). MYB TFs are key factors in the regulation pathways that control development, metabolism and response to biotic and abiotic stress [74]. Concerning their role in the drought tolerance, Seo et al. [75] reported that a R2R3-type MYB TF (MYB96) regulated drought stress response by integrating ABA and auxin signals. The putative MYB124 observed in the DeepSuperSAGE data (MYB124_TD22175) could be, along with MYB88, generating regular stomatal patterning, as in Arabidopsis [76], optimizing gas exchange and guard cell ion transport.

### Response to Oxidative Stress

Reactive oxygen species (ROS) production is a unifying commonality in a large number of abiotic stresses [77]. The redox-modulated changes are main events in cellular responses since ROS may help stress perception, but also damage the cell due to oxidation of membranes and other cellular components [78]. Responsive genes in such situations are, therefore, relevant to the maintenance of cellular homeostasis in adverse situations. The heat map based on the “response to oxidative stress” GO category and UR unitags in the approach I, compared with the approaches II and III, presented a set of UR unitags (approaches I and III), probably acting in the physiological behavior differentiation showed by the accessions, since these unitags were absent or n.s. in the sensitive accession (approach II, Cluster 1, Figure 7B; Table S7). One of the highest expressed modulated unitag (TD46995; FC_I_ = 110.4, FC_III_ = 110.4) was a putative peroxidase (PER). PER is an enzyme with oxidoreductase function that oxidizes a vast array of compounds (hydrogen donors) in the presence of H_2_O_2_. Like other enzymes from the ROS group, PER is a “ROS Scavenging Enzyme”. ROS scavenging increases the level of antioxidant enzymes, contributing to salt tolerance in different plants, including soybean [79]. This is in consonance with Zhang and Kirkhan [80] that observed an increase of peroxidase activity associated to the water deficit response. 

### Differential Response of the Accessions Based on Biological Processes (BP)

**Figure 8:**
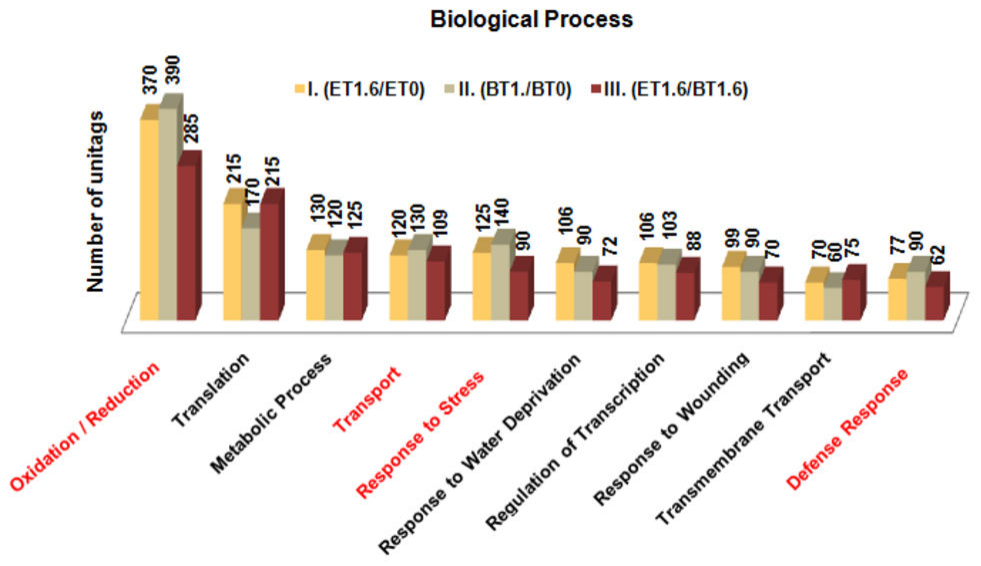
Number of UR unitags characterized by Gene Ontology (“Biological Process” subcategories), considering several contrasts*. UR: up-regulated; ^*^Tolerant accession under stress versus respective control (I); Sensitive accession under stress versus respective control (II) and Tolerant accession vs. Sensitive accession (both under stress; III).

A sample of the differential behavior between the studied accessions can be observed in the Figure 8, representing some BP subcategories with UR unitags observed in the approaches I, II and III. Considering six among 10 analyzed subcategories [“translation” (GO: 0006412), “metabolic process” (GO: 0008152), “response to water deprivation” (GO: 0009414), “regulation of transcription” (GO: 0045449), “response to wounding” (GO: 0009611), “transmembrane transport” (GO: 0055085)], the number of UR unitags in the tolerant accession (approach I) was larger than that in the sensitive one (approach II), indicating that, in those subcategories, the tolerant accession recruited and differentially expressed a larger number of unique transcripts. Also based on those subcategories, from the approach III it was clear that some UR unitags belonging to the tolerant accession were also up-regulated in relation to the sensitive accession, both under stress. From the quantitative point of view, the approach III revealed how many unitags were up-regulated in the tolerant accession, in relation to the sensitive one (both under stress). Here, the subcategory “transmembrane transport” had the largest number of UR unitags in the approach III, as compared to approach I. At a first glance, the results seemed to be incoherent. However, to the set of common UR unitags from approaches I and III it is necessary to include the constitutively expressed unitags from the approach I, as these tags are indeed up-regulated, when compared to the contrast III (both accessions under stress). 

On the other hand, in four subcategories [“oxidation reduction” (GO: 0055114), “transport” (GO: 0006810), “response to stress” (GO: 0006950), “defense response” (GO: 0006952)], a larger absolute number of UR unitags expressed by the sensitive accession (approach II), when compared to the tolerant one (approach I), demonstrated for those subcategories that the sensitive accession recruited and induced more unitags (Figure 8). Despite of this higher number of inducted unitags by the sensitive accession, in these subcategories, the overexpression by the tolerant accession in relation to the counterpart sensitive one, when both under stress, were demonstrated (approach III, Figure 8), pointing to a higher transcriptional efficiency of the tolerant over the sensitive after the stress. In short, the transcripts pool from the tolerant comparing with the sensitive accession, varied in both, quantitative and qualitatively aspects.

**Figure 9:**
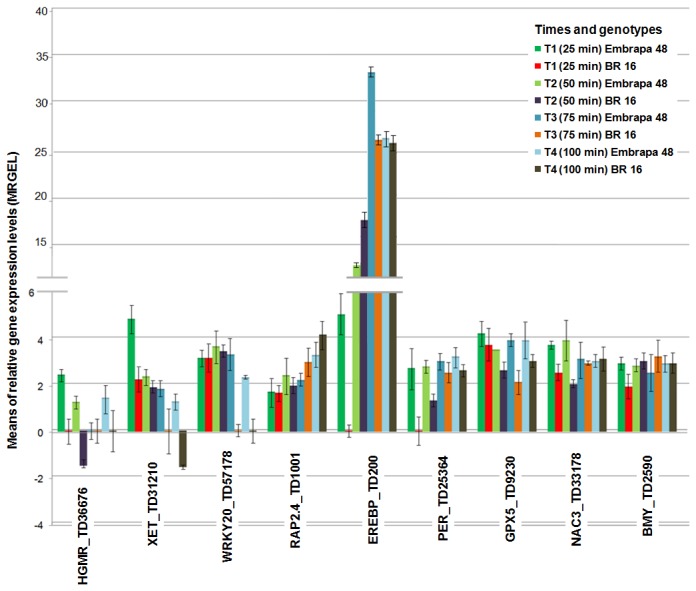
RT-qPCR of the unitags measured at the appropriate sample time using REST2009 software.

### Expression Analysis of Unitags in Contrasting Accessions by RT-qPCR

 The strategy to generate two DeepSuperSAGE libraries for each accession [negative control and bulk of samples gathering different times of stress imposition (25, 50, 75, 100, 125 and 150 min) reduced the number of libraries and became economically more realistic the project, but in turn also became more difficult to analyze the expression over the times sampled. The use of the RT-qPCR method provided the opportunity to integrate the differential expression of the candidate gene to the temporal variant opening of the bulked sample, based on expression in the times: 25, 50, 75, 100 min. The hereby studied nine genes (Table S8), covering contrasting and similar accession responses, present a concise overview of how transcriptional orchestration works in the analyzed condition, helping the understanding of the plant physiological behavior of each accession, and presenting the way that a transcript population changes over time in the addressed situation.

 Regarding the unitags showing different response between the accessions (UR in the tolerant and DR or n.s. in the sensitive contrasts in relation to the appropriate negative control), those validated by RT-qPCR were:

a) 3-hydroxy-3-methylglutaryl coenzyme A reductase [HMGR, EC 1.1.1.34; soybean gene model Glyma11g09330.1; unitag TD36676 (FC_tolerant_ = 24.0, FC_sensitive_ = -9.8)].
  The HMGR enzyme acts in the metabolism of isoprenoids, also called terpenoids. In plants, terpenoids show variation in structure and function, covering besides isoprenols (essential to biomembranes), also hormones, carotenoids and clorophyllins (photosynthetic pigments), among others [81]. Terpenoids can be synthesized by two mechanisms: desoxyxylulose 5-phosphate/2-C-methyl-D-erythritol 4 phosphate pathway (also called DPX pathway), widespread in eubacteria, and Mevalonate (MVA) pathway, prevalent in archaea and eukaryotes [82]. The HMGR enzyme catalyzes a key regulatory step of the MVA pathway, being modulated by various endogenous and external stimuli [83], including phytohormones, calcium, calmodulin, light, wounding, elicitor treatment, and pathogen attack [84]. Recently, Yang et al. [85] demonstrated in *Savia miltiorrhiza* hairy roots that HMGR mRNA levels and the enzyme activity were stimulated by abscisic acid (ABA) and methyl jasmonate (MJ), hormones known to be involved in the water deficit response, as well as polyethylene glycol (PEG), a compound that mimic drought stress effects in plants. The studied accessions differed regarding the transcriptional regulation of the unitag TD36676, in the course of the tested times (Figure 9), with the tolerant accession showing overexpression (*p* < 0.05) at all sampled times (MRGEL: 1.2 to 2.4; Figure 9), compared to the appropriate negative control (T0). Exception occurred for T3, in which the expression did not change (Figure 9). The sensitive accession, in turn, did not show differential expression at times T1, T3, T4, whereas, in T2, the expression decreased in regard to the sensitive negative control (MRGEL: - 1.5; Figure 9). Therefore, a role of this gene together with the MVA pathway in root dehydration differential response showed by the tolerant accession is likely.
b) Xyloglucan endotransglycosylase [XET, EC 2.4.1.207; soybean gene model Glyma13g38040.1; unitag TD31210 (FC_tolerant_ = 28.8, FC_sensitive_ = -6.8)].
  The XET or XTH enzyme acts in processes covering plant cell wall modifications. During cellular expansion, XET enhances the extensibility of the cell wall by cleaving xyloglucan at the xyloglucan–cellulose network presented in the plant cell wall [86,87]. Under water deficit conditions, the plant upper part growth inhibition and maintenance of root growth are often associated, in a well adaptive mechanism. However, in dry soils conditions, plant roots tend to grow seeking water richer zones. Despite XET relationship to plant cell wall strengthening processes [88], studies have shown a positive correlation with root elongation [89,90] and also of other plant organs [91]. The RT-qPCR results related to TD31210 unitag showed different behavior in both contrasting accessions as shown in Figure 9. The tolerant accession presented overexpression at all the analyzed stress times (MRGEL: 1.3 to 4.8 times, relative to T0; Figure 9). On the other hand, the sensitive one failed to keep this overexpression during the same studied times. Expression modulation for this accession (MRGEL: 1.9 to 2.2; Figure 9) occurred only in the first two times (25 and 50 min; Figure 9). In the following times (75 and 100 min), the expression was not significant or showed suppression (MRGEL: -1.6; Figure 9). Thus, the results suggest that radicular growth and soil remodeling may be involved in the tolerance observed in Embrapa 48 (tolerant accession), enabling the reestablishment of the proper functioning of its physiology.
c) Transcription Factor WRKY20 [soybean gene model Glyma05g36970.1; TD57178 unitag (FC_tolerant_ = 21.6, FC_sensitive_ = -4.4)].
  The main steps in plant tolerance to adverse environmental conditions process are stress condition perception, signal transduction, activation and regulation of stress responsive genes. The two previous steps requiring greater efforts at the transcriptional level, with a large portion of the plant genomics capacity driven by TFs. Soybean has 5,671 putative TFs, distributed in 63 families, which equates to 12.2% of the 46,430 predicted soybean protein-coding loci [25], while Arabidopsis and rice genomes code more than 2,100 and 2,300 TFs respectively [92]. Among TFs, WRKY is one of the largest families of plant transcriptional regulators modulating plant processes [93], also in plant stress responses. In rice, for instance, OsWRKY11 overexpression (under the control of HSP101 promoter) led to enhance drought tolerance [94]. Recently, Luo et al. [95] observed that the expression of wild soybean WRKY20 in Arabidopsis enhances drought tolerance and regulates ABA signalling. The differential behavior of the analyzed accessions for the expression (RT-qPCR) based on the TD57178 unitag was evident. The tolerant accession kept its induction over all the stress times tested (25, 50, 75, 100 min) modulating the expression (MRGEL) 2.3 to 3.6 times in relation to T0 (Figure 9); the sensitive accession, in turn, showed overexpression only in the early time points (25 and 50, MRGEL: 3.1 and 3.4, respectively, Figure 9). According to Chen et al. [96] the strict control and fine-tuning of WRKY proteins during plant stress responses contribute to the installation of complex signaling networks, highlighting the importance of WRKY proteins in plant abiotic stress response.

The unitags with similar regulation in both accessions under stress (Figure 9) allowed six genes to be RT-qPCR validated:

a) NAC3 transcription factor [NAC3; soybean gene model Glyma06g38410.1; unitag TD33178 (FC_tolerant_ = 33.6, FC_sensitive_ = 10.6); cluster 1 (gene expression heat map Figure 6A); Table S4].
  Under stress conditions, plants do not induce only gene transcriptions that operate in cellular protection, namely, enzyme coding genes and other functional proteins, but they also produce the regulatory transcripts that act in the transduction of signals from their perception organs. In this context are the transcription factors (TF) coding genes. Among the plant-specific transcription factors, NAC (NAM, ATAF, CUC) proteins constitute one of the largest families, present in a wide range of land plants [97]. The NAC was the most represented in the expression cluster “Response to hormonal stimulus” (Figure 6A). Specifically, the TD33178 unitag (NAC3 isoform) showed large modulation for both accessions (Figure 6A; Table S4); this being induced at all analyzed time intervals [tolerant accession (MRGEL: 3.0 to 3.9; sensitive accession: MRGEL: 2.0 to 3.1; Figure 9). This TF participation in the tolerance process to abiotic stresses has been demonstrated. Liu et al. [98] obtained tobacco transgenic lines transformed with *AhNAC3* (from peanut), and those showed hyper-resistance to dehydration and drought stresses and accumulated more proline and less superoxide anion (O_2_^−^) than wild type under dehydration and drought conditions. They also observed that four functional genes, superoxide dismutase, pyrroline-5-carboxylate synthetase, late embryogenic abundant proteins, and early response to drought 10, were induced in the transgenic lines, been suggested that NAC3 improves water stress tolerance by increasing superoxide scavenging and promoting the accumulation of various protective molecules.
b) AP2 (Apetala2) / ERF family, also called AP2/EREBP [99,100], presented the soybean gene models Glyma13g01930.1 and Glyma16g27950.1 associated, respectively, to the unitags TD1001 (FC_tolerant_ = 7.2; FC_sensitive_ = 6.0) and TD200 (FC_tolerant_ = 22.0; FC_sensitive_= 21.0).
  The AP2/ERF (EREBP) is a plant-specific TF large family that shares a well-conserved DNA-binding domain, comprising AP2, RAV, EREBP subfamilies, with the EREBP subfamily subdivided into DREB (Dehydration-responsive element-binding) or A subgroup and the ERF (Ethylene response factor) or B subgroup [101]. The up-regulation of those unitags observed in both accessions, in relation to the expression in the appropriate unstressed controls, suggests a conservative action even in contrasting accessions. Concerning the TD1001 unitag, the level of expression was similar for both accessions, considering each time evaluated, showing differential expression since 25 min after stress (Figure 9). Additionally, the unitag expression level in both accessions was smaller than those observed in TD200 (Figure 9). BLASTn analysis based on the RefSeq_RNA database (NCBI) revealed that Glyma13g01930.1 represents, specifically, an FT-type RAP2-4 (data not shown). RAP2-4 is a TF AP2/DREB-type, which belongs to EREBP subfamily. This TF was down-regulated by light but up-regulated by salt and drought stresses, in Arabidopsis [102]. Recently, Rae et al. [103] investigated the expression and function of RAP2-4B and RAP2-4 (both DREB TFs) using microarray-based transcriptional profiling of double knockout and overexpression lines. Expression analysis of stressed and control plants revealed both genes highly expressed in stems and roots and differentially induced in response to cold, dehydration and osmotic stress. The same authors also concluded that RAP2-4 is a probable significant aquaporin co-expression network regulator during the early phase of dehydration response. During that study, six aquaporin genes – from which three (*AtPIP2; 1*, *AtPIP2;2* and *AtPIP2;3*) from the PIP group and three (*AtTIP1;1*, *AtTIP2;2* and *At*TIP 2;3) from the TIP group – were down-regulated in the double knockout line and consequently up-regulated in the appropriate overexpression line [103]. In relation to the TD200 unitag (Glyma16g27950.1; annotated as a TF AP2/ERF, EREBP subfamily), the tolerant accession response revealing a faster response (25 min) than the sensitive accession (50 min; Figure 9). Also, in general, the average gene expression level presented by the tolerant accession was higher than the observed in the sensitive (Figure 9) also considering each analyzed time point. BLAST2Seq analysis performed to gather similarity between Glyma16g27950.1and Glyma13g01930.1 transcripts, since they belong to the same EREBP subfamily, did not show significance (data not shown). As mentioned before, this TF subfamily comprises DREBs and ERFs [101]. In soybean, the overexpression of a DREB homologous gene (GmDREB2) activated expression of downstream genes in transgenic Arabidopsis, resulting in enhanced tolerance to drought and high-salt stresses, without plant growth retardation [104]. Besides, its overexpression in tobacco resulted in higher proline content rates compared to wild type plants under drought condition [104]. ERFs also respond to drought tolerance. In soybean, GmERF3, a member of this subfamily, showed its expression induced by biotic stress [soybean mosaic virus, SMV] and abiotic stresses, such as high salinity, drought and hormones (ABA, SA, JA and ET) [105]. Aditionally, osmoregulation is among the known ERF-associated mechanisms. The overexpression of GmERF3 in transgenic tobacco led to higher levels of free proline and soluble carbohydrates compared to wild-type plants under drought conditions [105].
c) Β-amilase [BMY; soybean gene model Glyma15g10480.1; unitag TD2590 (FC_tolerant_ = 4.0, FC_sensitive_ = 3.5); cluster 1, gene expression heat map Figure 6B; Table S5].
  As mentioned before, it has been suggested that β-amylases act in the cellular osmotic regulation, when the plant is exposed to drought [65]. According to Ocampo and Robles [106] osmotic adjustment is the plant capacity to increase its solute concentration in leaves, roots and other organs responding to dehydration. This leads to the maintenance of the turgor pressure when the plant water potential declines, being crucial to the support of several biochemical and physiological processes [107]. In this study, one of the most abundant transcript classes in the gene expression heat map “Response to water” comprised 16 induced β-amylase isoforms in the tolerant accession and repressed or n.s. in the sensitive one (Figure 3B; Table S5). EST anchoring the TD2590 unitag after primers design and the respective RT-qPCR validation confirmed induction expression by both accessions, since the beginning of stress imposition (25 min) until the end time (100 min) [tolerant accession MRGEL: 2.5 to 2.9; sensitive accession: MRGEL: 1.9 to 3.2; Figure 9]. Such similar regulation in contrasting accessions, suggests the BMY importance in soybean root dehydration stress response.
d) Glutathione Peroxidase 5 [GPX5, EC 1.11.1.9; soybean gene model Glyma11g02630.1; unitag TD9230 (FC_tolerant_ = 16.9, FC_sensitive_= 12.8); gene expression heat map Figure 7B; Table S7].
  Plant survival in non-favorable growth conditions depends on its ability of stress perception, stimulus propagation and its physiology adaptation to new situations. As it was mentioned in the previous section (“Response to Oxidative Stress”) modulation of genes related to the cell redox status modulation is of utmost importance once such molecules act in the stress perception; however their excess presence damage cell structure [78]. By analyzing the active components of this response to oxidative stress and the gene expression heat map “Response to Oxidative Stress” (Figure 7B, Table S7), it was noticed that GPX and PER (see next target) were the most abundant enzymes. The TD9230 unitag, a potential GPX5 (Figure 7B; Table S7), after RT-qPCR validation, confirmed induction in both accessions, for all the analyzed time intervals, it being more modulated in the tolerant accession than in the sensitive (Figure 9). Yoshimura et al. [108] generated transgenic tobacco plants expressing a GPX-like protein in the cytosol (TcGPX) or chloroplasts (TpGPX). The transgenic plants showed increased tolerance to oxidative stress caused by application of methylviologen (MV: 50 µM) under moderate light intensity (200 μE m^-2^ sec^-1^), chilling stress under high light intensity (4 °C, 1000 μE m^-2^ sec^-1^), or salt stress (250 mM NaCl). In the transgenic plants the capacity of the photosynthetic and antioxidative systems remained higher than those of wild-type plants under chilling or salt stress.
e) Peroxidase [PER, EC 1.11.1.7; soybean gene model Glyma20g31190.1; unitag TD25364 (FC_tolerant_ = 33.6, FC_sensitive_= 10.6); gene expression heat map Figure 7B; Table S7].
  As mentioned before, PER was one of the most active components responding to oxidative stress (Figure 7B, Table S7). The unitag TD25364, a potential PER, presented induction (RT-qPCR; Figure 9) in both accessions. However, the tolerant accession response was faster, inducing PER since the time interval of 25 min, than the sensitive accession that only began to respond at the 50 min time interval (Figure 9). The quick response of the ROS scavenging associated machinery is of utter importance to plant organisms exposed to non-favorable growth conditions since it confers adaptative advantages to the organisms that behave adopting such transcriptional strategy. In this way, the tolerant accession would be more effective in this transcriptional response to the generated oxidative stress.

## Conclusions

This work provides novel genomic resources to support soybean approaches aiming to increase drought tolerance. A global evaluation of the soybean transcriptome under root dehydration stress using DeepSuperSAGE and high throughput sequencing allowed the identification of 1,127 unitags exclusively overexpressed in the stress-tolerant accession, many of them with considerable expression fold changes as compared to the tolerant negative control. Some of these were non-annotated unitags (209) only characterized by gene ontology terms using the EST anchoring the unitag. Other non-annotated induced unitags (272) showed “no hits”; these unknown transcripts were probably not yet associated to drought. Both groups comprise potential targets for further evaluation, validation and transgenesis. Also, some up-regulated unitags could be associated with important categories recognized by their role in plant abiotic stress response (e.g. “response to hormone stimulus”, “response to water”, “response to salt stress” and “response to oxidative stress”), revealing that the response to water deficit in both accessions recruited a repertoire of different genes, from the quantitative and qualitative point of views, with a higher number of induced genes as compared to those repressed. Additionally, data validation by RT-qPCR revealed an accession-specific transcriptome reprogramming detected 25 minutes after stress imposition, highlighting not only the effective responses associated to the tolerant accession, but also the non-efficient responses considering the sensitive accession.

## Materials and Methods

### Biological Material, Experimental Design and Stress Application

For root dehydration treatment, soybean (*G. max*) accessions ‘Embrapa 48’ (drought-tolerant) and ‘BR 16’ (drought-sensitive) [109] were grown in a greenhouse at Embrapa-Soybean station (Londrina, Brazil) using an aerated hydroponic system in 30 L plastic containers with pH 6.6-balanced nutrient solution as described by Kulcheski et al. [110]. Briefly, seeds were pre-germinated on moist filter paper in the dark at 25°C ± 1°C and in 65% ± 5% relative humidity. Plantlets were then placed in polystyrene supports, so the roots of the seedlings were fully immersed in the nutrient solution. Each seedling tray was maintained in a greenhouse at 25°C ± 2°C and in 60% ± 5% relative humidity under natural daylight (photosynthetic photon flux density (PPFD) = 1.5 × 10^3^ μmoles m^-2^ s^-1^, equivalent to 8.93 × 10^4^ lux) for 12 h/day. After 15 days, seedlings with the first trifoliate leaf fully developed (V2 developmental stage) [111] were submitted to different root dehydration periods, when the nutrient solution was removed from each plastic container where the roots were kept, in the tray, in the dark, without nutrient solution or water for 0 minutes (negative control) or 25 (T1), 50 (T2), 75 (T3), 100 (T4), 125 (T5), 150 minutes (T6). At the end of each period, the roots of the seedlings were immediately frozen in liquid nitrogen and stored at -80°C until RNA extraction. The experimental design was a factorial (accession × root dehydration times) with three replicates. Each replicate composed of five plantlets sampled in bulk. To avoid the impact of volatile compounds, each treatment was carried out in isolated spaces presenting the same growing conditions.

### RNA Extraction and Generation of DeepSuperSAGE Libraries

Total RNA was extracted of each treatment using the Plant RNeasy (Qiagen) kit, taking equimolar RNA quantities of each sample for bulk composition. Four DeepSuperSAGE libraries were generated with the bulks or the control RNA samples: ET1-6 (root dehydration-tolerant accession after stress – bulk of six times), BT1-6 (root dehydration-sensitive accession after stress – bulk of six times), ET0 (tolerant accession, negative control) and BT0 (sensitive accession, negative control). DeepSuperSAGE libraries were generated according to the procedures described by Matsumura et al. [112], under the guidance of GenXPro GmbH (Frankfurt, Germany) technical staff, with posterior SOLEXA sequencing of the tags. The data presented here can be downloaded from the Genosoja project (http://bioinfo03.ibi.unicamp.br/soybean/) [113]. 

### Statistical Analysis and Unitag-Gene Annotation

Tags (26 bp) were analyzed to identify unique tags (unitags) and those differentially expressed (*p* < 0.05), based on Poisson statistics developed by Audic and Claverie [114], as implemented in DiscoverySpace (v.4.01) software [115]. The singlets (tags sequenced only once) were excluded from the present evaluation. Unitags were annotated by BLASTn [116] against nucleotide sequences from following databases: (1) NCBI (the Plant Reference Sequence Database – RefSeq, and a limited dbEST file with ESTs from genera *Cicer* and *Pisum*; National Center for Biological Information, accessed in October 2012 [117]); (2) Kyoto Encyclopedia of Genes and Genomes, KEGG (ESTs from *Lotus japonicus*; *G. max*; *Vigna unguiculata*; *Phaseolus vulgaris*; *P. coccineus*; *Medicago truncatula*; *Arachis hypogaea* and *A. thaliana*; accessed in October 2012 [118]; (3) Resource for Plant Comparative Genomics, PlantGDB [119] (plant mRNAs multifasta file); (4) Plant Gene Indices / Gene Index Project (PHVGI, release 3-1; PCGI, release 1; GMGI, release 15; MTGI, release 9; LJGI, release 5 [10]); (5) Soybean Phytozome V5.0 (Glyma1 cDNA dataset [27]); (6) Nord*EST*: cowpea ESTs from Brazilian Nord*EST* network clustered with ESTs from the HarvEST-cowpea project [120]. The clusters and singlets were previously annotated by BLASTx (e-value cut off e^-10^) against the UniProtKB/Swiss-Prot database [121]. The BLASTn alignments (unitag-hit) with e-values of 0.001 or less and scores higher than 42, reflecting unitag-EST alignments tolerating a maximum of a single mismatch (TSM) were identified among the plus/plus alignments without mismatches regarding the four first bases CATG, to guarantee the integrity of the unitag. Besides the BLASTn analysis of unitags against ESTs, the soybean genome available in the Phytozome database (http://www.phytozome.org/) was also used in order to anchor unitags (TSM alignments) and to complement the analysis. 

### Gene Ontology of ESTs Anchoring DeepSuperSAGE Unitags

Multifasta file comprising the ESTs related to the unitags alignments (TSM) was analyzed by a local BLASTx using the UniProtKB/Swiss-Prot database and *e-value* cut-off e^-10^. The result imported by the software BLAST2GO v.2.4.4 [122] allowed the GO-mapping step. The GO terms in a data matrix together with the previously annotation results enabled data filtering and searches by keyword in a spreadsheet file.

### Keyword Search and Tag-Gene Annotation

Keyword searches performed on the original EST annotations included all BLASTn results and databases. Searches carried out on GO terms tried to confirm identities. The choice for best unitag-hit (EST) considered three consecutive rounds of redundancy elimination: (i) hits with inadequate/limited gene description and no GO term available; (ii) hits with only adequate description or only GO terms available and (iii) hits with adequate description and GO terms available. In each elimination round, only the best alignment (higher score, alignment size and identity) for each unitag, presenting (i) an adequate described soybean hit or (ii) in the absence of that, the best described hit from a soybean related species, suggested by Doyle and Luckow [123] or (iii) in the absence of both, an adequate described hit from another angiosperm, remained as the most informative ones.

### The Fold Change Estimation, the Heat Maps and Venn Diagram

Values reflecting expression data (*p-value* and up- or down-regulation regarding each unitag) were associated to the data matrix together with the respective unitag annotation, GO terms, the normalized frequencies in the libraries and the fold change values (FC). FC values comprised the ratio (R) of the normalized frequencies of one unitag in the contrast of two libraries, where the “zero” frequency was replaced by “one”. When R > 1 the FC were immediately considered and when R < 1 the FC = - 1/R. Negative FC values indicated repressed unitags. To generate heat maps considering different comparisons, differentially expressed unitags were hierarchically clustered (HAC) with support of the Cluster 3.0 (v.1.1.4r3) software [124] using default parameters and FC values as input data. The lateral dendrograms were generated using the TreeView software [125]. Finally, the Venn diagrams were generated with assistance of the software Venny [126].

### RT-qPCR Analyses

In order to substantiate the DeepSuperSAGE expression, nine selected unitags were validated by RT-qPCR. Unitags were selected based in their annotation, expression differentially regulated by the accessions and expressive FC values. Then, cDNAs related to the selected unitags were used for primers development, using the tool QuantPrime (http://www.quantprime.de/) and default parameters. The selected transcripts involved transcription factors [AP2 / ERF (EREBP), WRKY and NAC family], xyloglucan endotransglycosylase,3-hydroxy-3-methylglutaryl coenzyme A reductase 4, peroxidase, glutathione peroxidase 5 and β-amilase (Table S8).

Considering that DeepSuperSAGE and RT-qPCR are different methods, the expression levels observed with these procedures were not expected to be similar. So, to validate the DeepSuperSAGE data, the samples were not pooled for the RT-qPCR analysis, as they were for DeepSuperSAGE, and it was considered an agreement between the two approaches, when at least in one time point in RT-qPCR, similar results to DeepSuperSAGE (*p* < 0.05) were demonstrated. For this purpose, cDNA synthesis was achieved using total RNA extracted with Trizol^®^ reagent (Invitrogen) and the QuantiTec^®^ Reverse Transcription Kit (Qiagen); both according to the manufacturer’s instructions. RT-qPCR analyses were performed in a 7300 Real Time System (Applied Biosystems) thermocycler and the Platinum^®^ SYBR^®^ Green qPCR SuperMix UDG (Invitrogen). The reactions conditions were 50°C for 2 min, 95°C for 2 min, 45 cycles at 95°C for 15 s, 62°C for 30 s, and 72°C for 30 s; data were collected in the exponential phase of the RT-qPCR. The formula *E* = [10^−1/slope^] − 1 was applied to calculate the reaction efficiency. For each time point (0, 25, 50, 75, 100 min under root dehydration stress, three biological replicates, each with three technical replicates, were analyzed. Results were captured by the Sequence Detection program (Perkin Elmer) and analyzed by the Relative Expression Software Toll (REST) version 2.0.7 [127]. *Gmβ-actin* and *GmRNA18S* were used as reference genes for normalization [128]. Primers sequences, their efficiencies in RT-qPCR reactions and expected amplicons (bp) for the selected target genes are showed in Table S8.

All relative quantification was assessed using REST software 2009 [127,128], REST Standard, using the pair-wise fixed randomization test with 2,000 permutations.

## Supporting Information

Table S1 **Ca^2+^-binding proteins [calcium-dependent protein kinase (CDPK), calmodulin (CaM), and calcineurin B-like protein (CBL)] differentially expressed in at least one treatment.** Unitags associated to CDPKs, CaM and CBLs, their normalized frequencies, *p*-value [114], fold changes (FC_I_: tolerant accession under stress *vs* tolerant accession control; FC_II_: sensitive accession under stress *vs* sensitive accession control), regulation (in different approaches), identification, appropriate annotated EST, reference data bank, gene acronym and score regarding each unitag-EST alignment. *GMGI: *Glycine max* Gene Index (Plant Gene Indices / Gene Index Project Database); GDB: PlantGDB database; KEGG: Kyoto Encyclopedia of Genes and Genomes Database; PHVGI: *Phaseolus vulgaris* Gene Index (Plant Gene Indices / Gene Index Project Database) ^1^.ET1.6, tolerant accession under stress library; ET0, tolerant accession control library ^2^.BT1.6, sensitive accession under stress library; BT0, sensitive accession control library. UR: up-regulated; DR: down-regulated; n.s.: not significant (*p* < 0.05); (−) unitag not expressed.
(XLS)

Table S2 **Dehydrin transcripts differentially expressed in at least one treatment.** Unitags associated to dehydrins, their normalized frequencies, *p-value* [114], fold changes (FC_I_: tolerant accession under stress *vs* tolerant accession control; FC_II_: sensitive accession under stress *vs* sensitive accession control), their regulation (in different approaches), identification, appropriate annotated EST, reference data bank, gene acronym and score regarding each unitag-EST alignment. *GMGI: *Glycine max* Gene Index (Plant Gene Indices / Gene Index Project Database); GDB: PlantGDB database; MTGI: *Medicago truncatula* Gene Index (Plant Gene Indices / Gene Index Project Database) ^1^.ET1.6, tolerant accession under stress library; ET0, tolerant accession control library ^2^.BT1.6, sensitive accession under stress library; BT0, sensitive accession control library. UR: up-regulated; DR: down-regulated; n.s.: not significant (*p* < 0.05); (−) unitag not expressed.
(XLS)

Table S3 **ABC transporters differentially expressed in at least one treatment.** Unitags associated to ABC transporters, their normalized frequencies, *p-value* [114], fold changes (FC_I_: tolerant accession under stress *vs* tolerant accession control; FC_II_: sensitive accession under stress *vs* sensitive accession control), their regulation (in different approaches), identification, appropriate annotated EST, reference data bank, gene acronym and score regarding each unitag-EST alignment. *GMGI: *Glycine max* Gene Index (Plant Gene Indices / Gene Index Project Database); GDB: PlantGDB database; MTGI: *Medicago truncatula* Gene Index (Plant Gene Indices / Gene Index Project Database) ^1^.ET1.6, tolerant accession under stress library; ET0, tolerant accession control library ^2^.BT1.6, sensitive accession under stress library; BT0, sensitive accession control library. UR: up-regulated; DR: down-regulated; n.s.: not significant (*p* < 0.05); (−) unitag not expressed.
(XLS)

Table S4 **Data on the corresponding unitags presented in the heatmap regarding the GO category “Response to hormones” (Figure 6A).** Unitags associated to the term “Response to hormones”, their normalized frequencies, *p-value* [114], fold changes (FC_I_: tolerant accession under stress *vs* tolerant accession control; FC_II_: sensitive accession under stress *vs* sensitive accession control; FC_III_: tolerant accession under stress *vs* sensitive accession under stress), their regulation (in different approaches), identification, appropriate annotated EST, reference data bank, gene acronym and score regarding each unitag-EST alignment. *GMGI: *Glycine max* Gene Index, MTGI: *Medicago truncatula* Gene Index, *Phaseolus coccineus* Gene Index (Plant Gene Indices / Gene Index Project Database); GDB: PlantGDB database; KEGG: Kyoto Encyclopedia of Genes and Genomes Database ^1^.ET1.6, tolerant accession under stress library; ET0, tolerant accession control library ^2^.BT1.6, sensitive accession under stress library; BT0, sensitive accession control library. UR: up-regulated; DR: down-regulated; n.s.: not significant (*p* < 0.05); (−) unitag not expressed.
(XLS)

Table S5 **Data on the corresponding unitags presented in the heatmap regarding the GO category “Response to water” (Figure 6B).** Unitags associated to the term “Response to water”, their normalized frequencies, *p-value* [114], fold changes (FC_I_: tolerant accession under stress *vs* tolerant accession control; FC_II_: sensitive accession under stress *vs* sensitive accession control; FC_III_: tolerant accession under stress *vs* sensitive accession under stress), their regulation (in different approaches), identification, appropriate annotated EST, reference data bank, gene acronym and score regarding each unitag-EST alignment. *GMGI: *Glycine max* Gene Index (Plant Gene Indices / Gene Index Project Database); GDB: PlantGDB database; KEGG: Kyoto Encyclopedia of Genes and Genomes Database ^1^.ET1.6, tolerant accession under stress library; ET0, tolerant accession control library ^2^.BT1.6, sensitive accession under stress library; BT0, sensitive accession control library. UR: up-regulated; DR: down-regulated; n.s.: not significant (*p* < 0.05); (−) unitag not expressed.
(XLS)

Table S6 **Data on the corresponding unitags presented in the heatmap regarding the GO category “Response to salinity” (Figure 7A).** Unitags associated to the term “Response to salinity”, their normalized frequencies, *p-value* [114], fold changes (FC_I_: tolerant accession under stress *vs* tolerant accession control; FC_II_: sensitive accession under stress *vs* sensitive accession control; FC_III_: tolerant accession under stress *vs* sensitive accession under stress), their regulation (in different approaches), identification, appropriate annotated EST, reference data bank, gene acronym and score regarding each unitag-EST alignment. *GMGI: *Glycine max* Gene Index (Plant Gene Indices / Gene Index Project Database); GDB: PlantGDB database; KEGG: Kyoto Encyclopedia of Genes and Genomes Database ^1^.ET1.6, tolerant accession under stress library; ET0, tolerant accession control library ^2^.BT1.6, sensitive accession under stress library; BT0, sensitive accession control library. UR: up-regulated; DR: down-regulated; n.s.: not significant (*p* < 0.05); (−) unitag not expressed.
(XLS)

Table S7 **Data on the corresponding unitags presented in the heatmap regarding the GO category “Response to oxidative stress” (Figure 7B).** Unitags associated to the term “Response to oxidative stress”, their normalized frequencies, *p-value* [114], fold changes (FC_I_: tolerant accession under stress *vs* tolerant accession control; FC_II_: sensitive accession under stress *vs* sensitive accession control; FC_III_: tolerant accession under stress *vs* sensitive accession under stress), their regulation (in different approaches), identification, appropriate annotated EST, reference data bank, gene acronym and score regarding each unitag-EST alignment. *GMGI: *Glycine max* Gene Index (Plant Gene Indices / Gene Index Project Database); MTGI: *Medicago truncatula* Gene Index (Plant Gene Indices / Gene Index Project Database); KEGG: Kyoto Encyclopedia of Genes and Genomes Database ^1^.ET1.6, tolerant accession under stress library; ET0, tolerant accession control library ^2^.BT1.6, sensitive accession under stress library; BT0, sensitive accession control library. UR: up-regulated; DR: down-regulated; n.s.: not significant (*p* < 0.05); (−) unitag not expressed.
(XLS)

Table S8 **Target transcripts selected based on the DeepSuperSAGE differential expression for quantitative real-time amplification (RT-qPCR) including their gene acronym, primers sequences, amplicon length and primers efficiencies.**
(XLS)
